# Fear Memory Retrieval Is Associated With a Reduction in AMPA Receptor Density at Thalamic to Amygdala Intercalated Cell Synapses

**DOI:** 10.3389/fnsyn.2021.634558

**Published:** 2021-07-06

**Authors:** Anna Seewald, Sabine Schönherr, Heide Hörtnagl, Ingrid Ehrlich, Claudia Schmuckermair, Francesco Ferraguti

**Affiliations:** ^1^Department of Pharmacology, Medical University of Innsbruck, Innsbruck, Austria; ^2^Center for Integrative Neuroscience, Hertie Institute for Clinical Brain Research, University of Tübingen, Tübingen, Germany; ^3^Department of Neurobiology, Institute of Biomaterials and Biomolecular Systems, University of Stuttgart, Stuttgart, Germany

**Keywords:** fear conditioning, memory, freeze-fracture, plasticity, medial geniculate nucleus, glutamate receptor

## Abstract

The amygdala plays a crucial role in attaching emotional significance to environmental cues. Its intercalated cell masses (ITC) are tight clusters of GABAergic neurons, which are distributed around the basolateral amygdala complex. Distinct ITC clusters are involved in the acquisition and extinction of conditioned fear responses. Previously, we have shown that fear memory retrieval reduces the AMPA/NMDA ratio at thalamic afferents to ITC neurons within the dorsal medio-paracapsular cluster. Here, we investigate the molecular mechanisms underlying the fear-mediated reduction in the AMPA/NMDA ratio at these synapses and, in particular, whether specific changes in the synaptic density of AMPA receptors underlie the observed change. To this aim, we used a detergent-digested freeze-fracture replica immunolabeling technique (FRIL) approach that enables to visualize the spatial distribution of intrasynaptic AMPA receptors at high resolution. AMPA receptors were detected using an antibody raised against an epitope common to all AMPA subunits. To visualize thalamic inputs, we virally transduced the posterior thalamic complex with Channelrhodopsin 2-YFP, which is anterogradely transported along axons. Using face-matched replica, we confirmed that the postsynaptic elements were ITC neurons due to their prominent expression of μ-opioid receptors. With this approach, we show that, following auditory fear conditioning in mice, the formation and retrieval of fear memory is linked to a significant reduction in the density of AMPA receptors, particularly at spine synapses formed by inputs of the posterior intralaminar thalamic and medial geniculate nuclei onto identified ITC neurons. Our study is one of the few that has directly linked the regulation of AMPA receptor trafficking to memory processes in identified neuronal networks, by showing that fear-memory induced reduction in AMPA/NMDA ratio at thalamic-ITC synapses is associated with a reduced postsynaptic AMPA receptor density.

## Introduction

Associative learning is an adaptive process that allows an individual to predict events and requires long-lasting modifications in the strength of synaptic connections, generally known as synaptic plasticity (Martin et al., 2000; Malenka and Bear, 2004; Kessels and Malinow, 2009; Poo et al., 2016).

Pavlovian fear (threat) conditioning is one of the most prominently studied forms of associative learning (LeDoux, 2000; Maren, 2001), in which a neutral sensory stimulus (conditioned stimulus or CS) is repeatedly paired with an aversive stimulus (unconditioned stimulus or US; most commonly an electric shock), which leads to the formation of a strong CS-US association. In conditioned subjects, the presentation of the CS alone can trigger a fear response, that in rodents is commonly measured as freezing (LeDoux, 2000; Maren, 2001).

Somatosensory inputs arising from the neocortex and thalamus and carrying information about the CS and US converge in the lateral nucleus of the amygdala (LA) where persistent changes in synaptic transmission are linked to the encoding of the CS-US association and the storage of fear memories (Rogan et al., 1997; Pape and Paré, 2010). LA neurons then transfer the association *via* the basal and basomedial nuclei to the medial division of the central nucleus (CeA) to generate fear outputs (Maren and Quirk, 2004). This simple model has been recently challenged since other amygdaloid structures besides the LA, e.g., the lateral part of the CeA (CeL) and the intercalated cell masses of the amygdala (ITCs), were also shown to receive direct sensory information and undergo fear-related synaptic and cellular plasticity (Ciocchi et al., 2010; Haubensak et al., 2010; Li et al., 2013; Herry and Johansen, 2014; Asede et al., 2015; Barsy et al., 2020).

ITCs are a specialized group of tightly clustered GABAergic medium spiny neurons characterized by high μ-opioid receptor expression levels, exceeding those observed in the CeA and LA (Likhtik et al., 2008; Busti et al., 2011; Blaesse et al., 2015). The ITCs consist of several dispersed clusters located around the basolateral complex of the amygdala (BLA) (Millhouse, 1986; Busti et al., 2011). Two main ITC clusters are situated along the intermediate capsule, a dorsal medio-paracapsular (mpITC) and a larger ventrally located (vmITC) cluster (Millhouse, 1986; Kaoru et al., 2010; Busti et al., 2011; Hagihara et al., 2021). The vmITC is critically involved in the formation and/or recall of fear extinction memory (Likhtik et al., 2008; Amano et al., 2010; Hagihara et al., 2021). In contrast, the mpITC was found to be active during the expression of fear (Busti et al., 2011; Huang et al., 2014; Kwon et al., 2015; Hagihara et al., 2021). The vmITC has been proposed to be directly inhibited by the mpITC leading to disinhibition of the CeM and facilitation of fear responses (Royer et al., 2000; Busti et al., 2011). The engagement of different ITCs in distinct fear states may in part depend on their afferents, allowing a dual function to either facilitate or suppress conditioned fear responses (Busti et al., 2011; Duvarci and Paré, 2014).

We and others have recently demonstrated that efferents from the posterior intralaminar nuclei of the thalamus (PIN) and the medial subdivision of the medial geniculate nucleus (MGm), that convey pain- and auditory US- and CS-related information, innervate not only the LA but also the mpITC, whereas the vmITC is spared by this innervation (Asede et al., 2015; Strobel et al., 2015). The formation of both short- and long-term fear memory requires the potentiation of glutamatergic PIN/MGm to LA synapses (McKernan and Shinnick-Gallagher, 1997; Kim and Cho, 2017). We extended this by reporting a lasting decrease in pre- and post-synaptic strength upon fear memory retrieval at PIN/MGm to mpITC synapses (Asede et al., 2015).

Several studies have shown that the potentiation of LA synapses underlying fear memory formation involves a number of cellular and molecular mechanisms. These include modifications in transmitter release and gating properties of ionotropic glutamate receptors, such as α-amino-3-hydroxy-5-methyl-4-isoxazolepropionic acid (AMPA) and *N*-methyl-D-aspartate (NMDA) receptors (Huang and Kandel, 1998; Humeau et al., 2003; Rodrigues et al., 2004; Pape and Paré, 2010), but also an increase in the number of postsynaptic AMPA receptors (Rumpel et al., 2005; Yeh et al., 2006; Humeau et al., 2007; Migues et al., 2010). In particular, a sustained and long-lasting increase in AMPA to NMDA receptor EPSC amplitude ratio (A/N ratio) at thalamic to LA inputs was observed following auditory fear conditioning in mice (Clem and Huganir, 2010). The same behavioral paradigm, on the other hand, produced a significant decrease in A/N ratio at PIN/MGm to mpITC synapses upon fear memory retrieval (Asede et al., 2015).

Here, we hypothesize that the fear memory-associated reduction in the A/N ratio observed at PIN/MGm to mpITC synapses results from a change in the postsynaptic density of AMPA receptors. To investigate this at the level of single synapses, we took advantage of the Freeze-fracture replica immunolabeling technique (FRIL), which offers a two-dimensional planar view of synapses and thus allows the quantification of integral membrane proteins.

## Materials and Methods

### Animals

All procedures involving animals were performed according to methods approved by the Austrian Animal Experimentation Ethics Board (license: BMWF-66.011/004/wf/v/3b/2015 and BMWFW-66.011/0021-WF/V/3b/2016) and in compliance with the European convention for the protection of vertebrate animals used for experimental and other scientific purposes (ETS number 123). Every effort was taken to minimize animal suffering and the number of animals used. For this study, adult, 8–12 week old, male C57BL/6J mice (obtained at 7 weeks of age from Charles Rivers, Sulzfeld, Germany) were used. Mice were kept on a 12 h light/12 h dark cycle (lights off at 19:00). Mice had *ad libitum* access to standard chow and water and were housed in pairs of two.

### Viral Injections

For axonal tracing, the adenoviral vector AAV2/9-h.Syn-hChR2(H134R)-eYFP (AAV-ChR2-YFP) (Penn Vector Core, Philadelphia, PA, United States) was injected into the PIN/MGm at a concentration of 1.0 × 10^12^ gc/ml.

### Surgery

Stereotactic injection of the viral vector was carried out according to previously published procedures with minor modifications (Bosch et al., 2016; Schönherr et al., 2016). Eight week old mice were anaesthetized with an intraperitoneal (i.p.) injection of Ketasol/Xylazine (80/5 mg/kg). Anesthesia was maintained with 2% Sevofluran (SEVOrane, Abbvie) delivered *via* a face mask specifically designed for mice. For analgesia, 2 mg/kg Meloxicam (Metacam, Boehringer Ingelheim, Germany) were injected subcutaneously at the beginning of the surgery. Anesthetized mice were fixed in a stereotactic frame (DAVID KOPF Instruments, Tujunga, CA, United States) and injected bilaterally in the PIN/MGm at the following coordinates from bregma (in mm): posterior −2.70, lateral ± 1.60, ventral −3.60. Injections were performed with NEUROS Syringes (32G, 0.5 μl, HAMILTON, Reno, NV, United States), delivering a volume of 0.25 μl at an injection rate of 0.05 μl/min. Behavioral tests started 3 weeks after vector injection.

### Fear Conditioning

Fear conditioning and memory recall were performed on two consecutive days in 17 cm × 17 cm × 25 cm chambers (Ugo Basile, Comerio, Italy) with distinct floors (metal grid for footshock delivery vs. flat surface), wall patterns (transparent acrylic plastic vs. black and white stripes), lightning (100 lux white light vs. infrared light) and scents (70% EtOH vs. 1% acetic acid, also used as cleaning compound between subjects). For fear conditioning, mice were placed in the conditioning chamber for a total time of 10 min. Following a 120 s baseline period, mice were presented five times with a 30 s 80 dB white noise (WN) conditioned stimulus [CS, pseudorandom inter-stimulus interval (ISI) = 48–80 s] co-terminating with a 1s 0.7 mA footshock. The control group (CS only) underwent the same protocol, but footshock delivery was omitted. Fear memory retrieval was performed 24 h after fear conditioning and lasted 10 min. Following a 120 s baseline period, mice were presented four times with the CS (30 s, 80 dB, WN, pseudorandom ISI = 56–120 s). Freezing was measured as an index of fear by an automated procedure with the ANY-maze software (Stoelting Europe, Dublin, Ireland), using a freezing minimum duration threshold of 1 s, and manually cross-checked by a trained person.

### Histology

Thirty min after fear memory retrieval, animals were deeply anesthetized with thiopental sodium (150 mg/kg, i.p.) and transcardially perfused with a fixative [1% paraformaldehyde + 15% picric acid in 0.1 M phosphate-buffer (PB), pH 7.2–7.4]. Brains were immediately removed from the skull and placed in ice-cold 0.1 M PB. Coronal slices were cut with a vibratome (Leica VT1000S; Leica Microsystems, Vienna, Austria) at a thickness of 140 μm and collected in six well-dishes filled with 0.1 M PB. Sections containing the mpITC were then trimmed out under a stereomicroscope using an ophthalmic scalpel. Sections were cryoprotected with 30% glycerol in 0.1 M PB overnight at 6°C. Prior to the trimming, one slice/mouse containing the mpITC was mounted on an uncoated glass slide in 0.1 M PB, covered with a coverslip and the ChR2-YFP endogenous fluorescence was analyzed under an epifluorescence AxioImager M1 microscope (Carl Zeiss, Jena, Germany) to evaluate the pattern of distribution of ChR2-YFP-containing fibers. Digital images were taken using the Openlab software (Version 5.5.0, RRID:SCR_012158) through an Orca-ER CCD camera (Hamamatsu, Hamamatsu City, Japan). These slices were then demounted and the mpITC trimmed out as described above. Slices of the midbrain containing the injection site were similarly analyzed. Moreover, some slices were subsequently washed in Tris-buffered saline (TBS), incubated with a rabbit polyclonal anti-GFP primary antibody (Molecular Probes, Leiden, Netherlands, cat. no. A11122) diluted 1:1.000 in a solution containing 2% normal goat serum (NGS), 0.2% Triton X-100 in TBS (TBS-T) for 72 h at 6°C with constant shaking. Slices were then washed in TBS (three times for 10 min) and incubated overnight (6°C) with a donkey Alexa 488-conjugated anti-rabbit secondary antibody (1:1.000, Invitrogen, ThermoFisher Scientific, Waltham, MA, cat. no. A32790) in TBS-T and 2% NGS. To visualize general brain morphology, slices were counterstained with a DAPI solution (2 μg/ml, Sigma, cat. no. D-9564) for 4 min. Slices were then extensively washed in TBS, mounted onto gelatin-coated slides, and coverslipped with Vectashield (Vector Laboratory, Burlingame, CA, United States).

### Freeze-Fracture Replica Immunolabeling

The FRIL technique was originally introduced by Fujimoto (1995) and further developed by Shigemoto and coworkers (Tanaka et al., 2005). This study was carried out according to the procedures described in Schönherr et al. (2016). Briefly, trimmed sections were sandwiched between two copper carriers and frozen with a high-pressure freezing machine (HPM010; Bal-Tec, Balzers, Liechtenstein) followed by storage in liquid nitrogen. Carriers containing frozen sections were fractured and replicated using a freeze fracture machine (BAF060; Bal-Tec, Balzers, Liechtenstein). During this procedure, the plasma membrane breaks along the central hydrophobic core and, as a consequence, is split into two halves. The inner leaflet represents the protoplasmic halve of the membrane (P-face), whereas the outer leaflet faces the exoplasmic space (E-face). After fracturing, both faces were replicated through the deposition of platinum/carbon layers. At first, a carbon coat was applied at a speed of 0.1–0.2 nm/s to a thickness of 5 nm followed by a unidirectional platinum shadowing at a speed of 0.06–0.1 nm/s and to a thickness of 2 nm, with the platinum gun positioned at a 60° angle. Finally, a further 15 nm carbon layer was deposited at a speed of 0.3–0.5 nm/s. After thawing, the tissue attached to replicas was solubilized with shaking at 80°C overnight in the following solubilization buffer: 20% sucrose, 2.5% sodium dodecyl sulfate in 15 mM Tris buffer (TB), pH 8.3. Replicas were then transferred to a porcelain plate filled with fresh solubilization buffer and washed in TBS with 2.5% bovine serum albumin (BSA) for 5 min, followed by three washes, 10 min each, in 0.1% BSA-TBS. Non-specific binding sites were blocked by incubating the replicas in a blocking solution consisting of 5% BSA-TBS for 1 h. Primary antibodies were diluted in 2% BSA-TBS and then applied to the replicas. For each replicated specimen, one replica was incubated with the following primary antibodies: anti-pan-AMPA receptor (GluA1-4) diluted 1:200 (Frontier Institute, Hokkaido, Japan) and anti-green fluorescent protein (GFP) diluted 1:300 (Molecular Probes) that also detects YFP. The other corresponding replica was labeled with an antibody against μ-opioid receptors (dilution 1:500; ImmunoStar, Hudson, WI, United States). All incubations were performed in a 30 μl drop in a humid chamber at 15°C for 72 h. After the incubation in primary antibodies, replicas were washed in 0.1% BSA-TBS three times for 15 min and then transferred to a 2% BSA-TBS 30 μl drop, to which gold-conjugated secondary antibodies were added at a dilution of 1:30. To prevent confounds, AMPA receptors were visualized with antibodies conjugated to gold particles with a size of 5 nm, whereas ChR2-YFP with gold particles of 15 nm in diameter. Secondary antibodies against μ-opioid receptors were conjugated to 10 nm gold particles. Table 1 lists sources and concentrations of all primary and secondary antibodies used for FRIL experiments. Replicas were then mounted on pioloform-coated mesh (100-line parallel bars) copper grids and analyzed using a transmission electron microscope (Philips CM120) at 80 kV. Digital images were recorded with a Morada CCD camera (Olympus Soft Imaging Solutions GmbH, Münster, Germany) and the imaging software program iTEM (Olympus Soft Imaging Systems).

**TABLE 1:** List of primary and secondary antibodies used for FRIL.

Primary antibodies	Target molecule	Species	Dilution	Source	Catalog no.
Anti-GFP	Green fluorescent protein	Rabbit	1:300	Molecular probes	A11122
Anti-pan-AMPA	AMPA receptor (GluR1-4)	G. pig	1:200	Frontier Science	Pan-AMPA-GP-Af580-1
Anti-μ-opioid	μ-opioid receptor	Rabbit	1:500	Immunostar	24216
**Secondary antibodies**	Conjugated gold particle	Species	Dilution	Source	Batch no.
Anti-rabbit	10 nm	Donkey	1:30	Aurion	DAR-21211/2
Anti-rabbit	15 nm	Goat	1:30	BB International	7190
Anti-G. pig	5 nm	Goat	1:30	BB International	1175

### Measurement of Immunogold Particle Density

Synaptic density of AMPA receptors was calculated from electron micrographs of synapses taken at a magnification of 53 k. The postsynaptic membrane specialization (PSD) of glutamatergic synapses can be observed in replicas as a cluster of intramembrane particles (IMPs) on the E-face of the plasma membrane (Sandri et al., 1972; Masugi-Tokita and Shigemoto, 2007), and is often accompanied by the P-face of its presynaptic plasma membrane (Tarusawa et al., 2009). The synaptic area was delineated manually by following the perimeter of IMP clusters on the E-face using ImageJ Software (1.37 v, Java 1.6.0_65, Wayne Rasband, National Institutes of Health, United States). Postsynaptic specializations directly adjacent to the labeled plasma membrane of thalamic terminals were identified as targets of thalamic inputs. Receptor density was obtained by counting the number of gold particles within the synaptic area and is given as number of gold particles per μm^2^. The analyzed extrasynaptic area was the area of the plasma membrane adjacent to the IMP cluster and present within the 53 k digital images of the synapses. The pan-AMPA antibody was directed against extracellular epitopes and could thus be observed on the E-face of the plasma membrane, whereas GFP-labeling was detectable on the P-face of the plasma membrane. Non-specific labeling by the pan-AMPA antibody was determined on the P-face of surrounding structures and was subtracted from synaptic and extrasynaptic labeling densities.

As the mpITC is relatively small and did not fill the entire section, we took advantage of the fact that ITC neurons express high amounts of μ-opioid receptors in order to identify their postsynaptic processes. Therefore, every postsynaptic profile containing a synapse formed by a labeled thalamic terminal was confirmed to belong to a mpITC neuron by providing evidence of μ-opioid receptor labeling on the other membrane half (density > 7 gold particles/μm^2^).

### Gold Particle Analysis for Nanoscale Distribution of Synaptic AMPA Receptors

Spatial coordinates of the immunogold particles were extracted from electron micrographs and their arrangement was analyzed using the open source Python-based pipeline GoldExt (Szoboszlay et al., 2017). The nearest neighbor distance (NND) between gold particles within synapses was used to detect potential deviations from random distributions (50 random values calculated per synapse). For these analyses, only 80% of the sampled synapses were used, which, however, included all fully exposed synapses.

### Data Analysis

Data are presented as mean ± standard error of the mean (SEM). Comparison of multiple groups was done using one-way analysis of variance (ANOVA) followed by the Bonferroni *post hoc* test. Two-way ANOVA was used to analyse the effect of time and groups in the behavioral experiments. Cumulative distributions and correlations between synaptic area and number of gold particles were examined by the Kolmogorov–Smirnov and Pearson’s correlation coefficient tests. The Wilcoxon matched-pairs signed rank test was used to compare experimental with random NND distributions. Data were considered significant when *p* < 0.05. All statistical analyses were performed using the Prism 9 software (GraphPad, La Jolla, CA, United States).

## Results

**FIGURE 1:**
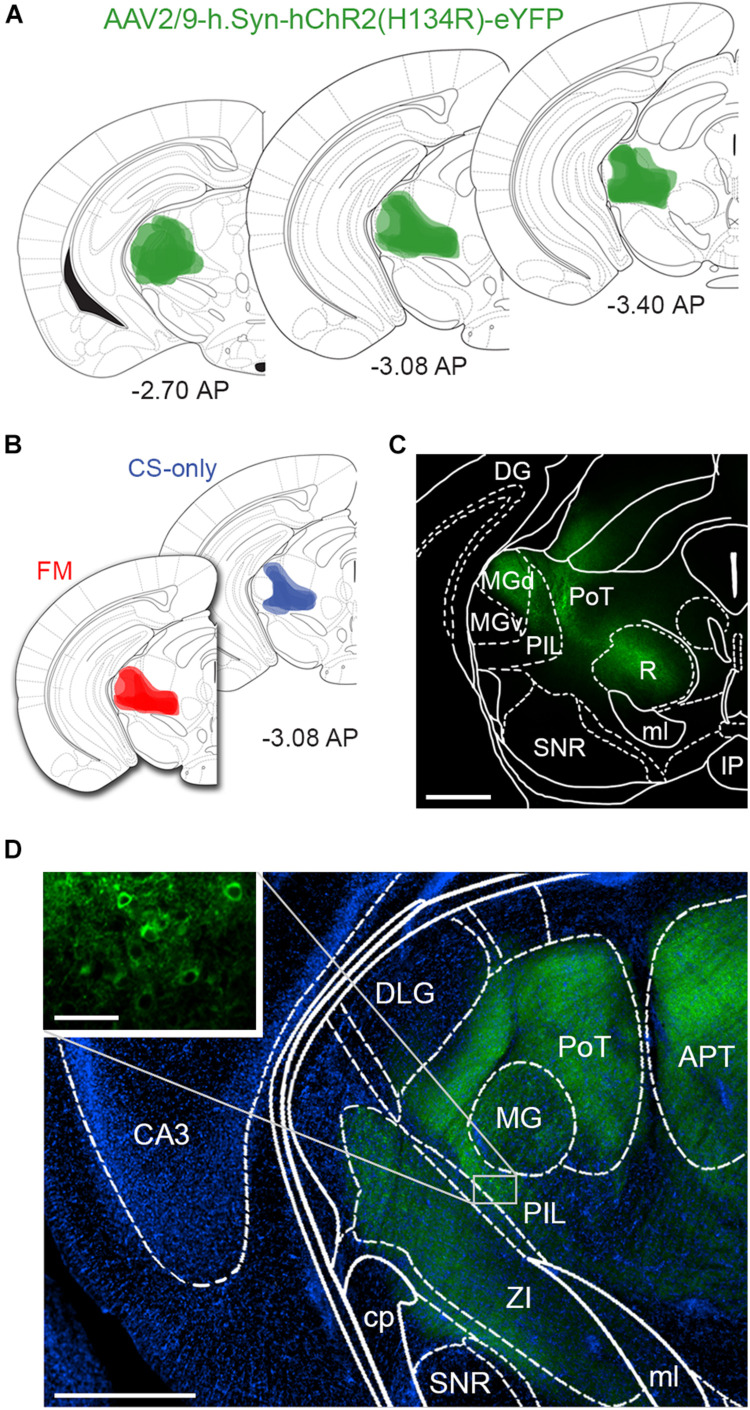
Intrathalamic injection sites of AAV-ChR2-YFP. **(A)** Schematic drawing of AAV-ChR2-YFP injection sites, where labeled neuronal cell bodies could be identified, at different rostro-caudal levels confirming that the microinjections were correctly placed into the PIN/MGm. Color intensity reflects the frequency of overlapping injection sites (*n* = 5 animals/group). Bregma levels are indicated in panels **(A,B)**. **(B)** Schematic drawings of AAV-ChR2-YFP injection sites in CS-only (blue) and fear conditioned mice (red). The two groups were largely similar in terms of injection sites, excluding a bias due to location differences in the microinjections. **(C)** Micrograph taken from a 140 μm thick coronal section of the midbrain showing ChR2-YFP endogenous fluorescence, overlaid with the brain atlas. Scale bar: 500 μm. **(D)** Immunodetection of ChR2-YFP transduced neurons and fibers (green); DAPI staining (blue) was used to reveal the macrostructure of the brain slice overlaid with the brain atlas. The inset shows somata of neurons in the PIL transduced with ChR2-YFP. The fluorescence signal in several brain areas close to the injection site, such as the APT and R, results from axonal fibers. Scale bar: 500 μm; inset: 50 μm. APT, anterior pretectal nucleus; CA3, field CA3 of hippocampus; cp, cerebral peduncle; DG, dentate gyrus; DLG, dorsal lateral geniculate nucleus; IP, interpeduncular nucleus; MG, medial geniculate nucleus; MGd, medial geniculate nucleus dorsal part; MGv, medial geniculate nucleus ventral part; ml, medial lemniscus; PAG, periaqueductal gray; PIL, posterior intralaminar thalamic nucleus; PoT, posterior thalamic nuclear group; R, red nucleus; SNR, Substantia Nigra pars reticulata; ZI, zona incerta.

**FIGURE 2:**
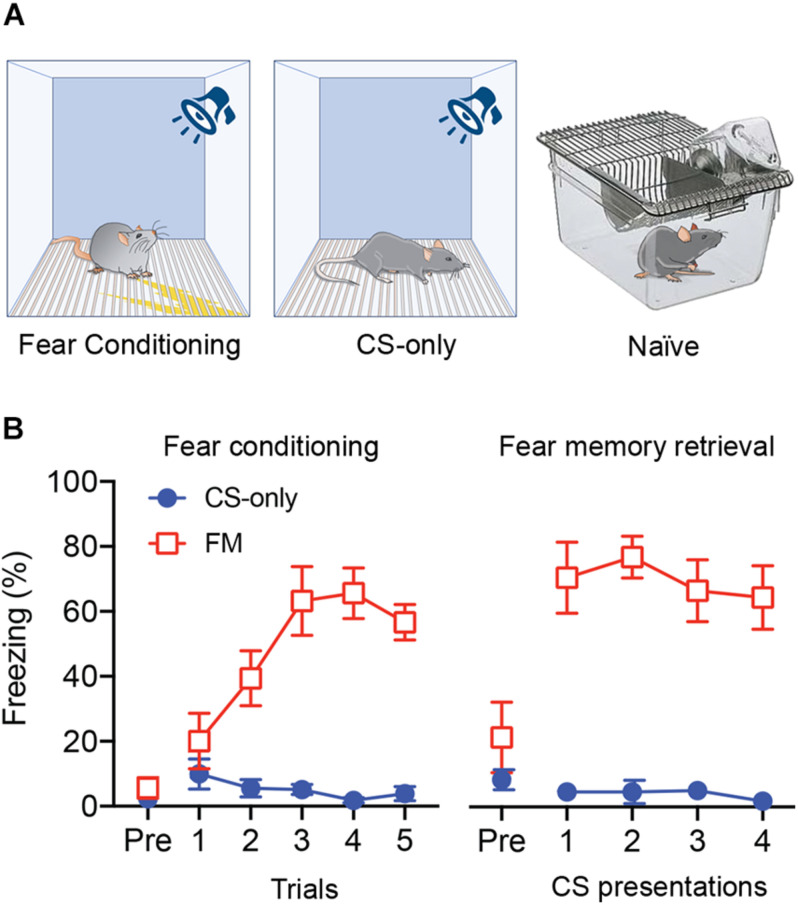
Auditory fear conditioning and fear memory retrieval. **(A)** Schematic diagram of the experimental groups. **(B)** Left: Average freezing responses of mice exposed to fear conditioning (FM) or only to the CS (CS-only) (*n* = 5 in each group) for each of the five CS-US pairing trials. Fear conditioned mice (FM) showed a progressive increase in freezing (2-way ANOVA, *p* < 0.0001) that was significantly higher compared to CS-only animals (*p* < 0.001). Right: When re-exposed, 24 h later, to the same CS for four times in a different context, fear conditioned mice (FM) showed higher freezing level compared to the pre-CS (Pre) period *(p* < 0.0001) and compared to CS-only mice *(p* < 0.001).

We stereotactically injected eight-week-old male mice (*n* = 15) with AAV-ChR2-YFP into the PIN/MGm (Figures 1A–D) and then randomly subdivided them into three groups (*n* = 5 each). Three weeks after the injection, one group was fear conditioned by subjecting the mice to five pairings of a neutral auditory CS co-terminating with a footshock (US), whereas a second group was exposed only to the CS in the conditioning chamber (Figure 2A). Animals in the third group (naïve) were always maintained in their home cage and did not undergo any behavioral manipulation (besides regular handling). Fear conditioned mice (FM group), but not the control group (CS-only), showed a progressive increase (2-way ANOVA, *p* < 0.001) in freezing (Figure 2B), demonstrating successful acquisition of a conditioned fear response. Subsequent to the behavioral testing, mice were returned to their home cages. Twenty-four hours later, they were re-exposed to four CS in a different context. Fear memory retrieval was clearly observed in previously fear conditioned mice, whereas mice in the CS-only group showed baseline freezing (Figure 2B). Thirty min after the end of the fear memory retrieval protocol, mice were perfused with fixative and brains processed for FRIL.

**FIGURE 3:**
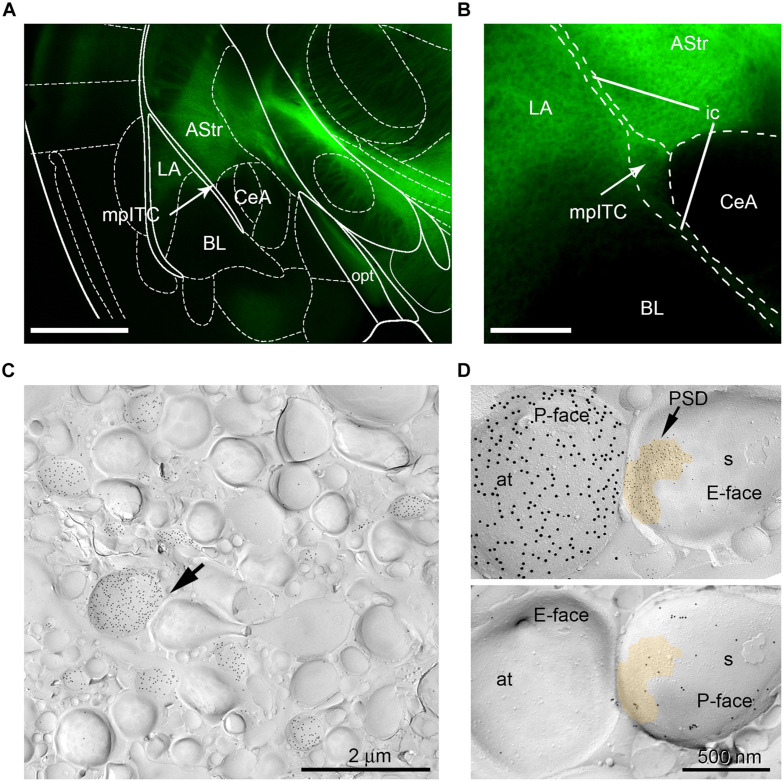
mpITC innervation by thalamic afferents from the PIN/MGm. **(A)** Fluorescence image of PIN/MGm efferent projections (green) innervating the amygdala. The LA, mpITC (indicated by the arrow) and AStr receive strong ChR2-YFP labeled inputs. Little to no fibers were observed in the BL and lateral CeA. Scale bar: 1 mm. **(B)** Higher magnification image of the PIN/MGm innervation of the mpITC. Scale bar: 250 μm. **(C)** Electron-micrograph of a mpITC replica from a CS-only animal. Labeling for ChR2-YFP (15 nm gold particles) on the P-face of axons originating from the PIN/MGm. Note the relatively high density of labeled axons. An axon terminal, indicated by the arrow, forms an asymmetric synapse with a spine. **(D)** Top: Higher magnification of the synapse marked (arrow) in panel **(C)**. The postsynaptic membrane specialization on the E-face shows a characteristic cluster of IMPs labeled with 5 nm gold particles revealing AMPA receptors. A pseudocolor is used to outline the postsynaptic specialization. The P-face of the axon terminal expresses a high density of ChR2-YFP (15 nm gold particles). Bottom: Corresponding P-face of the same spine labeled for μ-opioid receptors (10 nm gold particles). at, axon terminal; AStr, amygdalostriatal transition area; BL, basolateral nucleus of the amygdala; CeA, central nucleus of the amygdala; ic, internal capsule; LA, lateral nucleus of the amygdala; mpITC, dorsal medioparacapsular intercalated cluster; opt, optic tract; s, spine.

**FIGURE 4:**
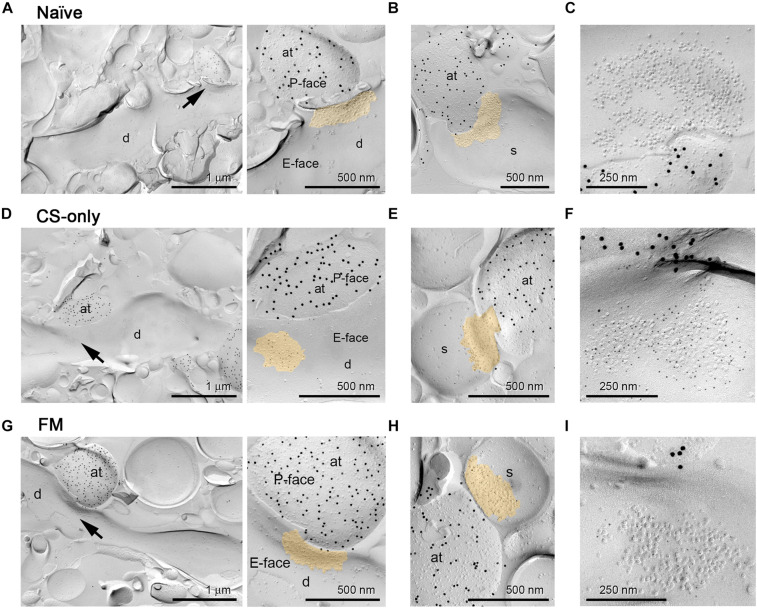
AMPA receptor immunogold labeling at PIN/MGm to mpITC synapses. Representative electron micrographs of PIN/MGm-mpITC shaft and spine synapses in all behavioral groups. **(A**–**C)** Naïve animals. **(A)** A ChR2-YFP expressing axon terminal (labeled with 15 nm gold particles on the P-face) forms a synaptic contact (arrow) with a spiny mpITC dendrite. An enlarged view of the pre- and post-synaptic membranes is shown on the right. **(B)** A ChR2-YFP expressing axon terminal (labeled with 15 nm gold particles) forms a synaptic contact with a dendritic spine of a mpITC neuron. **(C)** High magnification micrograph of an IMP cluster (postsynaptic specialization) on the E-face labeled for pan-AMPA receptors (5 nm gold particles). **(D**–**F)** CS-only animals. **(D)** A ChR2-YFP expressing axon terminal (labeled with 15 nm gold particles) forms a synaptic contact (arrow) with a spiny mpITC dendrite. An enlarged view of the pre- and post-synaptic membranes is shown on the right. **(E)** A ChR2-YFP expressing axon terminal (labeled with 15 nm gold particles) forms a synaptic contact with a dendritic spine of a mpITC neuron. **(F)** High magnification micrograph of an IMP cluster labeled for pan-AMPA receptors (5 nm gold particles). **(G**–**I)** FM animals. **(G)** A ChR2-YFP expressing axon terminal (labeled with 15 nm gold particles) forms a synaptic contact (arrow) with a spiny mpITC dendrite. An enlarged view of the pre- and post-synaptic membranes is shown on the right. **(H)** A ChR2-YFP expressing axon terminal (labeled with 15 nm gold particles) forms a synaptic contact with a dendritic spine of a mpITC neuron. **(I)** High magnification micrograph of an IMP cluster labeled for pan-AMPA receptors (5 nm gold particles). A pseudocolor is used to outline the postsynaptic specialization in A, B, D, E, G and H. Abbreviations: at, axon terminal; d, dendrite, s, dendritic spine.

**FIGURE 5:**
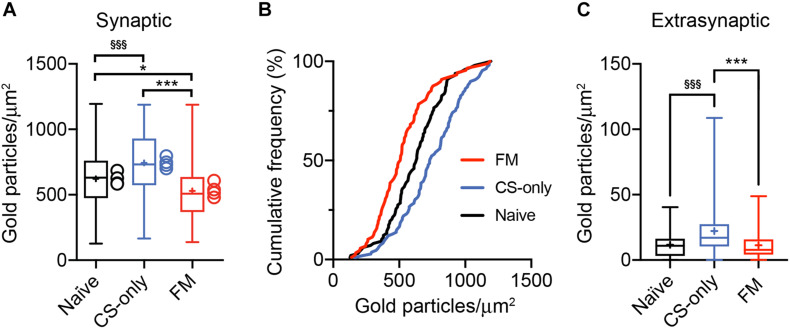
Reduction in AMPA receptor density at PIN/MGm to mpITC synapses upon fear memory retrieval. **(A)** Synapses from FM mice show a significant decrease in synaptic AMPA receptor density when compared to both CS-only (*p* < 0.0001, One-way ANOVA followed by the Bonferroni’s multiple comparison test) and naïve (*p* = 0.02) animals. Synapses (Naïve *n* = 98, CS-only *n* = 109, FM *n* = 88) were collected from 4 mice/group. CS-only animals showed a higher AMPA receptor density compared to naïve mice (*p* = 0.0005, Bonferroni’s multiple comparison test). Boxplots show the median (line inside the box), mean (+), 25th–75th percentiles (box edges) and minimum and maximum values (whiskers). The mean values for each mouse are shown as circles beside the box plots. **(B)** Significant left shift of the cumulative frequency distribution of synaptic AMPA receptor density in FM compared to the CS-only (*p* < 0.0001, Kolmogorov–Smirnov test) and naïve (*p* = 0.004) group. The cumulative frequency distribution of the AMPA receptor density is also significantly shifted to the right (*p* = 0.003) in CS-only compared to naïve synapses. **(C)** The density of extrasynaptic AMPA receptors is significantly higher in CS-only (*p* < 0.0001, One-way ANOVA followed by the Bonferroni’s multiple comparison test) compared to both naïve and FM animals. Boxplots show the median (line inside the box), mean (+), 25th–75th percentiles (box edges) and minimum and maximum values (whiskers). **(A,C)** Significantly higher than FM *p < 0.05, ***p < 0.001; Significantly higher than Naïve ^§§§^ *p* < 0.001.

Analysis of ChR2-YFP-transduced neurons revealed labeled somata inthe MGm and PIN, including the posterior thalamic nuclear group(PoT), posterior intralaminar nucleus (PIL), and peripeduncularnucleus (PP). ChR2-YFP-positive thalamic projections showed dense innervation of the LA, the amygdalostriatal transition area (AStr) and the mpITC cluster (Figures 3A,B), a pattern fully consistent with previous tracing studies (Turner and Herkenham, 1991; Linke et al., 2000; Asede et al., 2015; Bienvenu et al., 2015). At the electron microscope, intense gold immunolabeling for ChR2-YFP was observed on the P-face of thalamic axons (Figures 3C,D). AMPA receptors were detected using an antibody that recognizes all four subunits (GluA1-4). Spines and dendrites of ITC neurons were identified by their labeling for μ-opioid receptors on the corresponding replica P-face (Figure 3D), as these neurons express high-levels of these receptors (Likhtik et al., 2008; Busti et al., 2011; Blaesse et al., 2015; Schönherr et al., 2016). Synapses made by ChR2-YFP labeled axon terminals were sampled from replicas obtained from four animals per group (naïve = 123, CS-only = 188, FM = 200 synapses). However, for only about 58% of them (naïve = 98, CS-only = 109, FM = 88) the other halve of the plasma membrane could be identified in the corresponding replica labeled for μ-opioid receptors. This was mostly due to the fact that the region was placed over a mesh grid. The density of AMPA receptors was analyzed in both fully and partially exposed synapses (Figures 4A–I) by an experimenter blind to the behavioral treatment and limited to the confirmed ITC synapses. Data were pooled as they did not differ amongst animals within each group [One-way ANOVA; naïve: *F*(3,94) = 1.23, *p* = 0.30; CS-only: *F*(3,105) = 0.80, *p* = 0.49; FM: *F*(3,84) = 1.21, *p* = 0.31]. Upon fear memory retrieval, conditioned mice (FM group) showed a significant reduction in AMPA receptor density (529 ± 23.8 gold particles/μm^2^) when compared to both CS-only [743 ± 22.9 gold particles/μm^2^; One-way ANOVA, *F*(2, 292) = 22.00, *p* < 0.001, Bonferroni’s multiple comparisons test, *p* < 0.0001] and naïve (622 ± 21.8 gold particles/μm^2^; Bonferroni’s multiple comparisons test, *p* = 0.02) animals (Figure 5A). Thalamic to mpITC synapses in CS-only animals showed a higher AMPA receptor density than naïve mice (Bonferroni’s multiple comparisons test, *p* = 0.0005). Cumulative frequency distributions consistently revealed a significant shift to the left of the synaptic AMPA receptor density in FM vs. CS-only (Kolmogorov–Smirnov test, *p* < 0.0001) and naïve (Kolmogorov–Smirnov test, *p* = 0.004) animals, and a significant shift to the right (Kolmogorov–Smirnov test, *p* = 0.003) in synapses from the CS-only vs. naïve animals (Figure 5B). Given that AMPA receptors can shuttle between extrasynaptic and synaptic sites, we wanted to address if density changes were limited to PSDs, or also occurred in the proximity of synapses. By analyzing the AMPA receptor density in the extrasynaptic area proximal to the investigated synapses (up to ∼1 μm from the synapse outer border), we found that density differed significantly among groups [One-way ANOVA, *F*(2, 227) = 14.28, *p* < 0.0001]. The density of AMPA receptors was higher in the CS-only group (22.2 ± 2.06; Bonferroni’s multiple comparisons test, CS-only vs. both naïve and FM *p* < 0.0001), but similar in naïve (11.7 ± 1.21) and FM (11.3 ± 1.32) mice (Bonferroni’s multiple comparisons test, naïve vs. FM: *p* = 0.99; Figure 5C). A similar overall extrasynaptic area was analyzed for each group (Naïve: 43.56 μm^2^; CS-only: 31.13 μm^2^; FM: 33.59 μm^2^). Of note, the density of AMPA receptors in thalamo-mpITC synapses was approximately 60 times higher than in the adjacent extrasynaptic area (Figures 5A,C).

**FIGURE 6:**
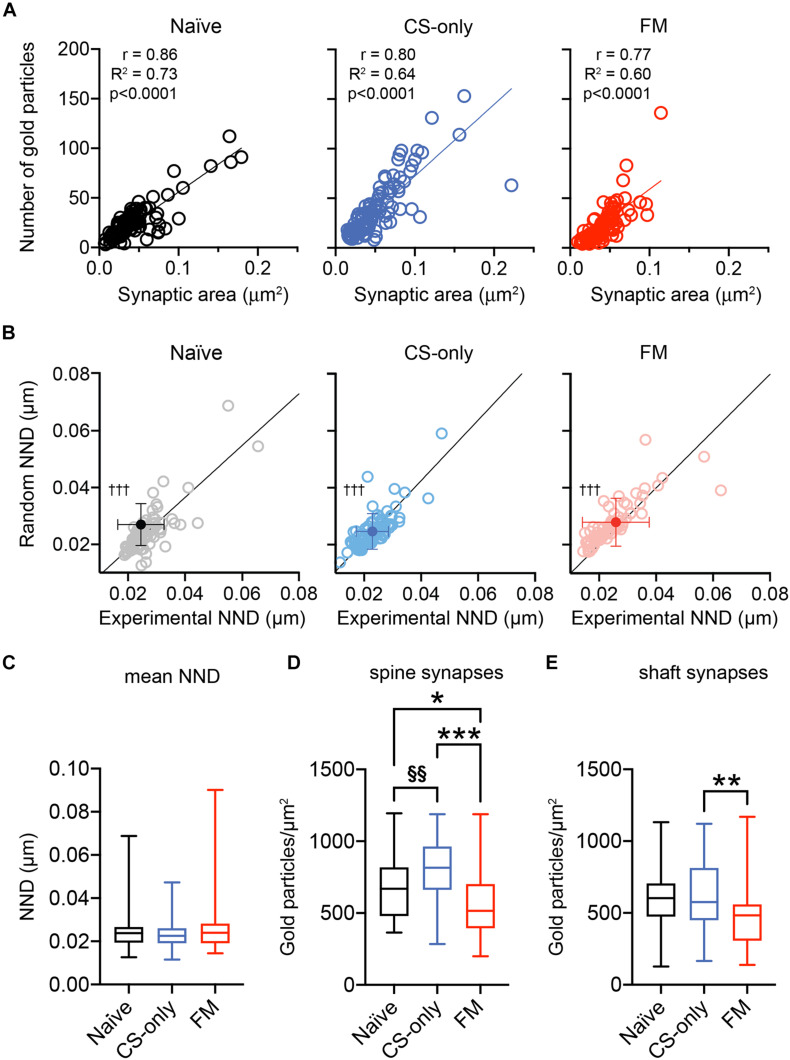
Nano-scale distribution of AMPA receptors and density at dendritic spine and shaft PIN/MGm-mpITC synapses. **(A)** PIN/MGm-mpITC synapses in all three experimental groups (FM, CS-only, and Naïve) show a highly significant positive correlation (Pearson’s correlation) between the number of gold particles detecting AMPA receptors and the synaptic area. The regression line is shown as a solid line passing through the origin. **(B)** Comparison of the experimentally determined mean nearest neighbor distance (NND) of intrasynaptic gold particles with random distributions (*n* = 50 for each PSD) reveals a significant difference from random distributions in all experimental groups (^†††^*p* < 0.001). **(C)** Boxplot summary of the NND obtained experimentally from PSDs of naïve (*n* = 80), CS-only (*n* = 89) and FM (*n* = 70) mice (*n* = 4 mice/group). Boxplots show the median (line inside the box), 25th–75th percentiles (box edges) and minimum and maximum values (whiskers). **(D**–**E)** Fear memory retrieval causes a marked decrease in AMPA receptor density in dendritic spine synapses when compared to both CS-only *(p* < 0.0001, One-way ANOVA followed by the Bonferroni’s multiple comparison test) and naïve *(p* = 0.038) animals. In FM mice, synapses made by PIN/MGm inputs onto dendrites had a lower density of AMPA receptors only in comparison to the CS-only (Bonferroni’s multiple comparisons test, *p* = 0.001) but not the naïve *(p* = 0.058) group. CS-only animals showed a higher AMPA receptor density than naïve mice, but only in spine (Bonferroni’s multiple comparisons test, *p* = 0.003) synapses. Synapses were collected from 4 animals/group. Boxplots show the median (line inside the box), 25th–75th percentiles (box edges) and minimum and maximum values (whiskers). Significantly higher than FM *p < 0.05, **p < 0.01, ***p < 0.001; Significantly higher than Naïve ^§§^*p* < 0.01.

Analysis of the correlation between the number of gold immunoparticles for AMPA receptors and the sampled area of individual synapses was found to be positively correlated in all three groups (Figure 6A), consistent with previous studies (Tanaka et al., 2005; Tarusawa et al., 2009; Fukazawa and Shigemoto, 2012; Xie et al., 2019). When the correlation coefficients were compared, after transforming the r values into z scores, no significant differences were found between the CS-only and the naïve (*z* test statistic: 1.38, *p* = 0.084) or FM (*z* test statistic: 0.538, *p* = 0.295) groups. On the other hand, the comparison of the correlations between FM and naïve animals significantly differed from each other (*z* test statistic: 1.829, *p* = 0.034). These data further support plasticity in the synaptic AMPA receptor content associated with fear memory retrieval. Next, we analyzed the intrasynaptic spatial arrangement of AMPA receptors using the open source software GoldExt (Szoboszlay et al., 2017). We measured the mean NND for each synapse and examined whether the population distribution differed from random arrangements. Since there was no difference in the calculated NND between fully and partially exposed synapses (Mann–Whitney test, naïve: synapses on dendrites *p* = 0.20, synapses on spines: *p* = 0.89; CS-only: synapses on dendrites *p* = 0.17, synapses on spines: *p* = 0.65; FM: synapses on dendrites *p* = 0.86, synapses on spines: *p* = 0.58), data were pooled. The distribution across the NND populations obtained from naïve, CS-only and FM mice significantly differed from random distributions (Wilcoxon matched-pairs signed rank test, *p* < 0.001 for all three groups; Figure 6B). Furthermore, the mean NND was similar [One-way ANOVA, *F(*2, 236) = 2.39, *p* = 0.09] among the three experimental groups (naïve: 0.024 ± 0.001, CS-only: 0.023 ± 0.001; FM: 0.026 ± 0.001 μm; Figure 6C). These findings suggest that synaptic AMPA receptors do not change their homogeneous, though clustered, intrasynaptic nano-arrangement as a consequence of sensory stimulation and/or fear memory retrieval.

Finally, as we found that thalamic afferents provide inputs onto both dendritic shafts (Figures 4A,D,G) and spines (Figures 4B,E,H), we wanted to understand if experience-induced changes (Figure 5A) could be more prominent, or differentially observed in one of these two synapse types. Therefore, we analyzed whether spine and shaft synapses differed in AMPA receptor density. In all three behavioral groups synapses on spines had a higher density of AMPA receptors [naïve: 673 ± 31.7 (*n* = 44); CS-only: 810 ± 25.0 (*n* = 70); FM: 566 ± 29.1 (*n* = 58) gold particles/μm^2^] compared to those on dendrites [naïve: 579 ± 29.1 (*n* = 54); CS-only: 622 ± 39.4 (*n* = 39); FM: 458 ± 38.1 (*n* = 30) gold particles/μm^2^], although only in CS-only and FM mice it reached statistical significance (Mann–Whitney test 2 tailed; naïve: *p* = 0.082; CS-only: *p* < 0.0001; FM: *p* = 0.046). For spine synapses, the FM group showed a marked reduction in AMPA receptor density [One-way ANOVA, *F*(2, 169) = 21.08, *p* < 0.0001] when compared to both CS-only (Bonferroni’s multiple comparisons test, *p* < 0.0001) and naïve (*p* = 0.038) animals (Figure 6D). In contrast, synapses on dendrites in FM mice showed a lower density of AMPA receptors [One-way ANOVA, *F*(2, 120) = 4.75, *p* < 0.010] only when compared to the CS-only (Bonferroni’s multiple comparisons test, synapses on spines: *p* = 0.001] but not the naïve (*p* = 0.058) group (Figure 6E). Remarkably, AMPA receptor density increased in synapses made by thalamic inputs in CS-only compared to naïve mice only for spine (Bonferroni’s multiple comparisons test, *p* = 0.003) but not for shaft synapses (Bonferroni’s multiple comparisons test *p* = 0.99). Taken together these data suggest that experience-dependent changes in AMPA receptor density in FM and CS-only groups are more prominently observed in dendritic spine than shaft synapses onto mpITCs.

## Discussion

We show here that following auditory fear conditioning in mice, the formation and retrieval of fear memory is linked to a significant reduction in the density of AMPA receptors in synapses made by PIN/MGm inputs onto identified mpITC neurons. Our findings suggest that the decrease in A/N ratio observed at the same synapses upon fear memory retrieval (Asede et al., 2015) results from an altered trafficking of AMPA receptors, and in particular their removal from the PSD of spine synapses. While many mechanisms can influence the A/N ratio, the addition and removal of AMPA receptors from synapses is one of the fundamental processes controlling synaptic efficacy during experience-dependent plasticity (Choquet and Triller, 2013; Huganir and Nicoll, 2013; Henley and Wilkinson, 2016; Choquet, 2018; Diering and Huganir, 2018). In addition, we have previously reported that fear memory retrieval is associated with an increase in paired pulse ratio (PPR) of the AMPA-EPSC at PIN/MGm-mpITC synapses (Asede et al., 2015). Despite changes in PPR have been typically viewed as a hallmark of presynaptic modulation of transmitter release, it has been suggested that postsynaptic AMPA receptor mobility could also influence PPR (Frischknecht et al., 2009; Choquet, 2018).

In the last decade, the development of the FRIL technique has considerably improved the ability to analyses the intrasynaptic distribution of signaling molecules (Masugi-Tokita and Shigemoto, 2007). One of the greatest advantages of this approach relies on its high detection sensitivity, for instance in comparison with the post-embedding immunogold technique. Indeed, a tight correlation between the number of immunogold particles detecting synaptic AMPA receptors and functional channels was observed in immature rat cerebellar Purkinje cell synapses (Tanaka et al., 2005). Although in our study we did not attempt to quantify the number of functionally active AMPA channels at PIN/MGm to mpITC synapses, we assume that it is highly correlated with the number of detected gold particles. AMPA receptor labeling at these synapses showed a tight positive correlation with the sampled synaptic area corroborating the view that this is a common feature of many excitatory synaptic connections in the central nervous system (Fukazawa and Shigemoto, 2012). The selective identification of PIN/MGm axon terminals in replicas was enabled by a novel approach that takes advantage of the anterograde transport of tagged opsins, such as ChR2-YFP, and their detection at defined presynaptic sites, since they are integral membrane proteins (Schönherr et al., 2016).

So far, two types of AMPA receptor intrasynaptic distributions have been described (reviewed in Fukazawa and Shigemoto, 2012): (1) a highly variable mosaic-type distribution, such as in corticogeniculate (Tarusawa et al., 2009) and CA3-CA1 synapses (Matsubara et al., 1996; Nusser et al., 1998), and (2) a homogeneous distribution with high density, such as in reticulogeniculate synapses (Tarusawa et al., 2009). The synapses made by PIN/MGm inputs onto mpITC neurons showed, despite a fairly wide density range (from 127 to 1,195 particles/μm^2^), a dense and relatively uniform pattern of distribution of AMPA receptors. However, the mean NND was significantly shorter than expected from random arrangements. PIN/MGm to mpITC synapses, therefore, appear to possess most of the features typical of the second type. This homogeneous AMPA receptor distribution might serve to ensure high transmission fidelity, as observed for several GABAergic interneurons. For example, synapses made with interneurons distributed throughout multiple layers of the hippocampus have a higher and less variable density of AMPA receptors compared to synapses made with spines of pyramidal neurons (Nusser et al., 1998). Likewise, a very dense and homogeneous AMPA receptor labeling was described for synapses made by parallel fibers with interneurons of the cerebellar cortex, unlike those made with Purkinje cell spines (Masugi-Tokita et al., 2007). Interestingly, it has been suggested that synaptic connections with high and homogeneous intrasynaptic distribution of AMPA receptors have different plastic properties than mosaic-type synapses, e.g., they could undergo more pronounced structural modifications of the postsynaptic density (Fukazawa and Shigemoto, 2012). Therefore, our observed reduction in AMPA receptor density linked to fear memory retrieval may also result, at least in part, from a synaptic enlargement. Because the full exposure of the postsynaptic specialization was relatively rare under our experimental conditions, fear-mediated changes in the synaptic area could not be analyzed. Future studies will have to address whether mpITC synapses made by PIN/MGm inputs undergo structural plasticity.

AMPA receptors are homo- and/or hetero-tetramers made from four subunits (GluA1-4). Synaptic AMPA receptors are predominantly combinations of GluA1 and GluA2 (Lu et al., 2009). Memory consolidation in the BLA is commonly associated with increased GluA1 and GluA2 subunit expression (Yeh et al., 2006; Ferrara et al., 2017). However, at thalamo-amygdala synapses alterations in GluA3 subunit expression have been reported (Radley et al., 2007). To circumvent confounds caused by different subunit compositions, we used a pan-AMPA antibody recognizing all four subunits. Hence, our data allow conclusions only about global changes of synaptic AMPA receptor density, but not specific alterations in subunit composition.

Our work revealed that synapses made by PIN/MGm inputs onto mpITC neurons have a significantly higher density of AMPA receptors when they are made with dendritic spines than shafts. This finding is consistent with a previous study showing that latero-capsular division neurons in the CeA have higher AMPA receptor density in spine compared to shaft synapses (Dong et al., 2010). Spine synapses are the most common type of excitatory synapses in both cortical and striatal-like structures and a large body of evidence demonstrated that they undergo structural and functional changes associated with synaptic plasticity (Sala and Segal, 2014; Segal, 2017). Conversely, little is known about shaft synapses. Dong et al. (2010) suggested that the difference in AMPA receptor density between spine and shaft synapses in the CeA was most likely related to different presynaptic inputs. This may be unlikely for mpITC neurons. Indeed, several studies reported that axons arising from the PIN/MGm form synapses with both spines and dendrites in the LA (Radley et al., 2007; Smith et al., 2019). We cannot, however, exclude that different neuronal populations within the PIN/MGm give rise to inputs preferentially targeting dendritic spines vs. shafts of mpITC neurons. Spine and shaft synapses may also vary in their molecular composition, which in turn could affect their plasticity and ability to cluster AMPA receptors (Mi et al., 2002; Bissen et al., 2019). Notably, we report that the decrease in AMPA receptor density following fear memory retrieval was more prominently observed at spine synapses. This new finding is consistent with the notion of a considerable ability of spine synapses to undergo plasticity. Alternatively, it could also be hypothesized that changes at spine synapses last longer than at shaft synapses, perhaps due to their tortuous geometry that slows down receptor diffusion, and consequently, the recovery from internalization (Ashby et al., 2006; Newpher and Ehlers, 2008). Surprisingly, we also observed a significant increase in the density of AMPA receptors in spine, but not shaft, synapses upon sensory stimulation in CS-only animals in comparison to naïve mice. This was paralleled also by an increased density in the extrasynaptic area. Indeed, mpITC neurons respond to a variety of auditory stimuli (Collins and Paré, 1999), which could lead to sensory-driven synaptic strengthening by recruitment of synaptic AMPA receptors. Previous studies have shown that exocytosis of intracellular vesicles harboring AMPA receptors occurs near the postsynaptic membrane (Ehlers, 2013; Wu et al., 2017). Therefore, a recruitment of synaptic AMPA receptors is consistent with a concurrent increase of these receptors in extrasynaptic areas neighboring postsynaptic specializations.

A remarkable feature of synapses made by PIN-MGm inputs is their divergent forms of plasticity depending on the postsynaptic targets, e.g., LA and mpITC neurons (Clem and Huganir, 2010; Asede et al., 2015). Although we do not know whether LA and mpITC neurons are contacted by axons of the same PIN-MGm neurons or independently by distinct neuronal populations, the first postulation appears to be the most parsimonious one. A factor that may influence this postsynaptic cell-dependent form of plasticity is the very different firing rate of these two classes of target neurons. While LA pyramidal neurons have a very low spontaneous firing rate (Paré and Gaudreau, 1996), ITC cells fire at higher rates in all behavioral states. The high spontaneous firing rates of mpITCs suggest that they provide a tonic inhibition onto their downstream targets, such as the vmITC, to promote fear expression (Collins and Paré, 1999; Royer et al., 2000; Busti et al., 2011). An additional intriguing feature of mpITC neurons is the long-term inverse heterosynaptic modification reported to occur after a particular input has undergone activity-dependent enhancement, which may be needed to the stabilization of total synaptic weights (Royer and Paré, 2003). A decrease in sensory input strength may, in turn, help to facilitate plasticity at inputs from activated LA neurons onto mpITC cells (Huang et al., 2014) and consequently fear learning. Therefore, an opposing plasticity at mpITC and LA synapses could serve to coordinate state-dependent changes of activity in these parallel fear circuits.

In conclusion, our study is one of the few that have attempted to link the regulation of AMPA receptor trafficking to memory processes in identified neuronal networks. In particular, we show that fear-memory induced reduction in A/N ratio at thalamic-mpITC synapses is associated with a reduced postsynaptic AMPA receptor density. However, we still do not know precisely how this is mediated, or how stable these synaptic changes are. Future investigations are needed to unravel other potential contributions to the fear-mediated plasticity of mpITC synapses, such as changes in AMPA receptor subunit configuration and/or NMDA receptor expression.

## Data Availability Statement

The raw data supporting the conclusions of this article will be made available by the authors, without undue reservation.

## Ethics Statement

The animal study was reviewed and approved by the Austrian Animal Experimentation Ethics Board.

## Author Contributions

AS, IE, CS, and FF conceived and designed the project. AS, SS, HH, CS, and FF were involved with experimental and analytical aspects of the manuscript. AS, CS, and FF wrote the manuscript. All authors commented on the manuscript.

## Conflict of Interest

The authors declare that the research was conducted in the absence of any commercial or financial relationships that could be construed as a potential conflict of interest.

## Abbreviations

AMPA: α-amino -3-hydroxy-5-methyl-4-isoxazolepropionic acid
A/N ratio: AMPA to NMDA receptor EPSC amplitude ratio
AAV-ChR2-YFP: AAV2/9-h.Syn-hChR2(H134R)-eYFP
BLA: basolateral complex of the amygdala
CS: conditioned stimulus
CeA: central nucleus of the amygdala
CeL: lateral part of the CeA
FM: fear memory
IMPs: intramembrane particles
ITCs: intercalated cell masses of the amygdala
LA: lateral nucleus of the amygdala
MGm: medial subdivision of the medial geniculate nucleus
mpITC: dorsal medio-paracapsular cluster
NMDA: *N*-methyl -D-aspartate
NND: nearest neighbor distance
PB: phosphate-buffer
PIN: posterior intralaminar nuclei of the thalamus
PPR: paired pulse ratio
PSD: postsynaptic membrane specialization
TBS: Tris-buffered saline
US: unconditioned stimulus
vmITC: ventral medial cluster.

## References

[B1] Amano T. Unal C. T. Paré D. (2010). Synaptic correlates of fear extinction in the amygdala. *Nat. Neurosci.* 13 489–494. 10.1038/nn.2499 20208529 PMC2847017

[B2] Asede D. Bosch D. Lüthi A. Ferraguti F. Ehrlich I. (2015). Sensory inputs to intercalated cells provide fear-learning modulated inhibition to the basolateral amygdala. *Neuron* 86 541–554. 10.1016/j.neuron.2015.03.008 25843406

[B3] Ashby M. C. Maier S. R. Nishimune A. Henley J. M. (2006). Lateral diffusion drives constitutive exchange of AMPA receptors at dendritic spines and is regulated by spine morphology. *J. Neurosci*. 26 7046–7055. 10.1523/JNEUROSCI.1235-06.2006 16807334 PMC6673929

[B4] Barsy B. Kocsis K. Magyar A. Babiczky A. Szabo M. Veres J. M. (2020). Associative and plastic thalamic signaling to the lateral amygdala controls fear behavior. *Nat. Neurosci.* 23 625–637. 10.1038/s41593-020-0620-z 32284608

[B5] Bienvenu T. C. Busti D. Micklem B. R. Mansouri M. Magill P. J. Ferraguti F. (2015). Large intercalated neurons of amygdala relay noxious sensory information. *J. Neurosci.* 35 2044–2057. 10.1523/JNEUROSCI.1323-14.2015 25653362 PMC4315833

[B6] Bissen D. Foss F. Acker-Palmer A. (2019). AMPA receptors and their minions: auxiliary proteins in AMPA receptor trafficking. *Cell. Mol. Life Sci.* 76 2133–2169. 10.1007/s00018-019-03068-7 30937469 PMC6502786

[B7] Blaesse P. Goedecke L. Bazelot M. Capogna M. Pape H. C. Jüngling K. (2015). μ-opioid receptor-mediated inhibition of intercalated neurons and effect on synaptic transmission to the central amygdala. *J. Neurosci.* 35 7317–7325. 10.1523/JNEUROSCI.0204-15.2015 25972162 PMC4429148

[B8] Bosch D. Asede D. Ehrlich I. (2016). Ex Vivo Optogenetic Dissection of Fear Circuits in Brain Slices. *J. Vis. Exp.* 2016:e53628. 10.3791/53628 27077317 PMC4841359

[B9] Busti D. Geracitano R. Whittle N. Dalezios Y. Mañko M. Kaufmann W. (2011). Different fear states engage distinct networks within the intercalated cell clusters of the amygdala. *J. Neurosci.* 31 5131–5144. 10.1523/JNEUROSCI.6100-10.2011 21451049 PMC6622967

[B10] Choquet D. (2018). Linking nanoscale dynamics of AMPA receptor organization to plasticity of excitatory synapses and learning. *J. Neurosci.* 38 9318–9329. 10.1523/JNEUROSCI.2119-18.2018 30381423 PMC6705996

[B11] Choquet D. Triller A. (2013). The dynamic synapse. *Neuron* 80 691–703. 10.1016/j.neuron.2013.10.013 24183020

[B12] Ciocchi S. Herry C. Grenier F. Wolff S. B. Letzkus J. J. Vlachos I. (2010). Encoding of conditioned fear in central amygdala inhibitory circuits. *Nature* 468 277–282. 10.1038/nature09559 21068837

[B13] Clem R. L. Huganir R. L. (2010). Calcium-permeable AMPA receptor dynamics mediate fear memory erasure. *Science* 330 1108–1112. 10.1126/science.1195298 21030604 PMC3001394

[B14] Collins D. R. Paré D. (1999). Spontaneous and evoked activity of intercalated amygdala neurons. *Eur. J Neurosci.* 11 3441–3448. 10.1046/j.1460-9568.1999.00763.x 10564352

[B15] Diering G. H. Huganir R. L. (2018). The AMPA Receptor Code of Synaptic Plasticity. *Neuron* 100 314–329. 10.1016/j.neuron.2018.10.018 30359599 PMC6214363

[B16] Dong Y.-L. Fukazawa Y. Wang W. Kamasawa N. Shigemoto R. (2010). Differential postsynaptic compartments in the laterocapsular division of the central nucleus of amygdala for afferents from the parabrachial nucleus and the basolateral nucleus in the rat. *J. Comp. Neurol.* 518 4771–4791. 10.1002/cne.22487 20963828

[B17] Duvarci S. Paré D. (2014). Amygdala microcircuits controlling learned fear. *Neuron* 82 966–980. 10.1016/j.neuron.2014.04.042 24908482 PMC4103014

[B18] Ehlers M. D. (2013). Dendritic trafficking for neuronal growth and plasticity. *Biochem. Soc. Trans.* 41 1365–1382. 10.1042/bst20130081 24256224

[B19] Ferrara N. C. Cullen P. K. Pullins S. P. Rotondo E. K. Helmstetter F. J. (2017). Input from the medial geniculate nucleus modulates amygdala encoding of fear memory discrimination. *Learn. Mem.* 24 414–421. 10.1101/lm.044131.116 28814467 PMC5580525

[B20] Frischknecht R. Heine M. Perrais D. Seidenbecher C. I. Choquet D. Gundelfinger E. D. (2009). The brain extracellular matrix limits lateral diffusion of AMPA receptors and modulates short-term synaptic plasticity. *Nat. Neurosci.* 12 897–904. 10.1038/nn.2338 19483686

[B21] Fujimoto K. (1995). Freeze-fracture replica electron microscopy combined with SDS digestion for cytochemical labeling of integral membrane proteins. Application to the immunogold labeling of intercellular junctional complexes. *J. Cell. Sci.* 108 3443–3449. 10.1242/jcs.108.11.3443 8586656

[B22] Fukazawa Y. Shigemoto R. (2012). Intra-synapse-type and inter-synapse-type relationships between synaptic size and AMPAR expression. *Curr. Opin. Neurobiol.* 22 446–452. 10.1016/j.conb.2012.01.006 22325858

[B23] Hagihara K. M. Bukalo O. Zeller M. Aksoy-Aksel A. Karalis N. Limoges A. (2021). Intercalated amygdala clusters orchestrate a switch in fear state. *Nature* 594 403–407. 10.1038/s41586-021-03593-1 Online ahead of print, 34040259 PMC8402941

[B24] Haubensak W. Kunwar P. S. Cai H. Ciocchi S. Wall N. R. Ponnusamy R. (2010). Genetic dissection of an amygdala microcircuit that gates conditioned fear. *Nature* 468 270–276. 10.1038/nature09553 21068836 PMC3597095

[B25] Henley J. M. Wilkinson K. A. (2016). Synaptic AMPA receptor composition in development, plasticity and disease. *Nat. Rev. Neurosci.* 17 337–350. 10.1038/nrn.2016.37 27080385

[B26] Herry C. Johansen J. P. (2014). Encoding of fear learning and memory in distributed neuronal circuits. *Nat. Neurosci.* 17 1644–1654. 10.1038/nn.3869 25413091

[B27] Huang C.-C. Chen C.-C. Liang Y.-C. Hsu K.-S. (2014). Long-term potentiation at excitatory synaptic inputs to the intercalated cell masses of the amygdala. *Int. J. Neuropsychopharmacol.* 17 1233–1242. 10.1017/S1461145714000133 24556032

[B28] Huang Y. Y. Kandel E. R. (1998). Postsynaptic induction and PKA-dependent expression of LTP in the lateral amygdala. *Neuron* 21 169–178. 10.1016/s0896-6273(00)80524-3 9697861

[B29] Huganir R. L. Nicoll R. A. (2013). AMPARs and synaptic plasticity: the last 25 years. *Neuron* 80 704–717. 10.1016/j.neuron.2013.10.025 24183021 PMC4195488

[B30] Humeau Y. Reisel D. Johnson A. W. Borchardt T. Jensen V. Gebhardt C. (2007). A pathway-specific function for different AMPA receptor subunits in amygdala long-term potentiation and fear conditioning. *J. Neurosci.* 27 10947–10956. 10.1523/JNEUROSCI.2603-07.2007 17928436 PMC6672841

[B31] Humeau Y. Shaban H. Bissiére S. Lüthi A. (2003). Presynaptic induction of heterosynaptic associative plasticity in the mammalian brain. *Nature* 426 841–845. 10.1038/nature02194 14685239

[B32] Kaoru T. Liu F. C. Ishida M. Oishi T. Hayashi M. Kitagawa M. (2010). Molecular characterization of the intercalated cell masses of the amygdala: implications for the relationship with the striatum. *Neuroscience* 166 220–230. 10.1016/j.neuroscience.2009.12.004 20004711

[B33] Kessels H. W. Malinow R. (2009). Synaptic AMPA receptor plasticity and behavior. *Neuron* 61 340–350. 10.1016/j.neuron.2009.01.015 19217372 PMC3917551

[B34] Kim W. B. Cho J. H. (2017). Encoding of discriminative fear memory by input-specific LTP in the amygdala. *Neuron* 95 1129–1146. 10.1016/j.neuron.2017.08.004 28823727

[B35] Kwon O. B. Lee J. H. Kim H. J. Lee S. Lee S. Jeong M. J. (2015). Dopamine Regulation of Amygdala Inhibitory Circuits for Expression of Learned Fear. *Neuron* 88 378–389. 10.1016/j.neuron.2015.09.001 26412489

[B36] LeDoux J. E. (2000). Emotion circuits in the brain. *Annu. Rev. Neurosci.* 23 155–184. 10.1146/annurev.neuro.23.1.155 10845062

[B37] Li H. Penzo M. A. Taniguchi H. Kopec C. D. Huang Z. J. Li B. (2013). Experience-dependent modification of a central amygdala fear circuit. *Nat. Neurosci.* 16 332–339. 10.1038/nn.3322 23354330 PMC3581751

[B38] Likhtik E. Popa D. Apergis-Schoute J. Fidacaro G. A. Paré D. (2008). Amygdala intercalated neurons are required for expression of fear extinction. *Nature* 454 642–645. 10.1038/nature07167 18615014 PMC2528060

[B39] Linke R. Braune G. Schwegler H. (2000). Differential projection of the posterior paralaminar thalamic nuclei to the amygdaloid complex in the rat. *Exp. Brain Res.* 134 520–532. 10.1007/s002210000475 11081834

[B40] Lu W. Shi Y. Jackson A. C. Bjorgan K. During M. J. Sprengel R. (2009). Subunit composition of synaptic AMPA receptors revealed by a single-cell genetic approach. *Neuron* 62 254–268. 10.1016/j.neuron.2009.02.027 19409270 PMC3632349

[B41] Malenka R. C. Bear M. F. (2004). LTP and LTD: an embarrassment of riches. *Neuron* 44 5–21. 10.1016/j.neuron.2004.09.012 15450156

[B42] Maren S. (2001). Neurobiology of Pavlovian fear conditioning. *Annu. Rev. Neurosci.* 24 897–931. 10.1146/annurev.neuro.24.1.897 11520922

[B43] Maren S. Quirk G. J. (2004). Neuronal signalling of fear memory. *Nat. Rev. Neurosci.* 5 844–852. 10.1038/nrn1535 15496862

[B44] Martin S. J. Grimwood P. D. Morris R. G. (2000). Synaptic plasticity and memory: an evaluation of the hypothesis. *Annu. Rev. Neurosci.* 23 649–711. 10.1146/annurev.neuro.23.1.649 10845078

[B45] Masugi-Tokita M. Shigemoto R. (2007). High-resolution quantitative visualisation of glutamate and GABA receptors at central synapses. *Curr. Opin. Neurobiol*. 17 387–393. 10.1016/j.conb.2007.04.012 17499496

[B46] Masugi-Tokita M. Tarusawa E. Watanabe M. Molnár E. Fujimoto K. Shigemoto R. (2007). Number and density of AMPA receptors in individual synapses in the rat cerebellum as revealed by SDS-digested freeze-fracture replica labeling. *J. Neurosci.* 27 2135–2144. 10.1523/JNEUROSCI.2861-06.2007 17314308 PMC6673557

[B47] Matsubara A. Laake J. H. Davanger S. Usami S. Ottersen O. P. (1996). Organization of AMPA receptor subunits at a glutamate synapse: a quantitative immunogold analysis of hair cell synapses in the rat organ of Corti. *J. Neurosci.* 16 4457–4467. 10.1523/JNEUROSCI.16-14-04457.1996 8699256 PMC6578857

[B48] McKernan M. G. Shinnick-Gallagher P. (1997). Fear conditioning induces a lasting potentiation of synaptic currents in vitro. *Nature* 390 607–611. 10.1038/37605 9403689

[B49] Mi R. Tang X. Sutter R. Xu D. Worley P. O’Brien R. J. (2002). Differing mechanisms for glutamate receptor aggregation on dendritic spines and shafts in cultured hippocampal neurons. *J. Neurosci.* 22 7606–7616. 10.1523/JNEUROSCI.22-17-07606.2002 12196584 PMC6757964

[B50] Migues P. V. Hardt O. Wu D. C. Gamache K. Sactor T. C. Wang Y. T. (2010). PKMζ maintains memories by regulating GluR2-dependent AMPA receptor trafficking. *Nat. Neurosci.* 13 630–634. 10.1038/nn.2531 20383136

[B51] Millhouse O. E. (1986). The intercalated cells of the amygdala. *J. Comp. Neurol.* 247 246–271. 10.1002/cne.902470209 2424941

[B52] Newpher T. M. Ehlers M. D. (2008). Glutamate receptor dynamics in dendritic microdomains. *Neuron* 58 472–497. 10.1016/j.neuron.2008.04.030 18498731 PMC2572138

[B53] Nusser Z. Lujan R. Laube G. Roberts J. D. Molnar E. Somogyi P. (1998). Cell type and pathway dependence of synaptic AMPA receptor number and variability in the hippocampus. *Neuron* 21 545–559. 10.1016/s0896-6273(00)80565-6 9768841

[B54] Pape H. C. Paré D. (2010). Plastic synaptic networks of the amygdala for the acquisition, expression, and extinction of conditioned fear. *Physiol. Rev.* 90 419–463. 10.1152/physrev.00037.2009 20393190 PMC2856122

[B55] Paré D. Gaudreau H. (1996). Projection cells and interneurons of the lateral and basolateral amygdala: distinct firing patterns and differential relation to theta and delta rhythms in conscious cats. *J. Neurosci.* 16 3334–3350. 10.1523/JNEUROSCI.16-10-03334.1996 8627370 PMC6579143

[B56] Poo M. M. Pignatelli M. Ryan T. J. Tonegawa S. Bonhoeffer T. Martin K. C. (2016). What is memory? The present state of the engram. *BMC Biol.* 14:40. 10.1186/s12915-016-0261-6 27197636 PMC4874022

[B57] Radley J. J. Farb C. R. He Y. Janssen W. G. Rodrigues S. M. Johnson L. R. (2007). Distribution of NMDA and AMPA receptor subunits at thalamo-amygdaloid dendritic spines. *Brain Res.* 1134 87–94. 10.1016/j.brainres.2006.11.045 17207780 PMC2359729

[B58] Rodrigues S. M. Farb C. R. Bauer E. P. LeDoux J. E. Schafe G. E. (2004). Pavlovian fear conditioning regulates Thr286 autophosphorylation of Ca2+/calmodulin-dependent protein kinase II at lateral amygdala synapses. *J. Neurosci.* 24 3281–3288. 10.1523/JNEUROSCI.5303-03.2004 15056707 PMC6730013

[B59] Rogan M. T. Stäubli U. V. LeDoux J. E. (1997). Fear conditioning induces associative long-term potentiation in the amygdala. *Nature* 390 604–607. 10.1038/37601 9403688

[B60] Royer S. Martina M. Paré D. (2000). Polarized synaptic interactions between intercalated neurons of the amygdala. *J. Neurophysiol.* 83 3509–3518. 10.1152/jn.2000.83.6.3509 10848566

[B61] Royer S. Paré D. (2003). Conservation of total synaptic weight through balanced synaptic depression and potentiation. *Nature* 422 518–522. 10.1038/nature01530 12673250

[B62] Rumpel S. LeDoux J. E. Zador A. Malinow R. (2005). Postsynaptic receptor trafficking underlying a form of associative learning. *Science* 308 83–88. 10.1126/science.1103944 15746389

[B63] Sala C. Segal M. (2014). Dendritic spines: the locus of structural and functional plasticity. *Physiol. Rev.* 94 141–188. 10.1152/physrev.00012.2013 24382885

[B64] Sandri C. Akert H. Livingston R. B. Moor H. (1972). Particle aggregations at specialized sites in freeze-etched postsynaptic membranes. *Brain Res.* 41 1–16. 10.1016/0006-8993(72)90612-9 5036038

[B65] Schönherr S. Seewald A. Kasugai Y. Bosch D. Ehrlich I. Ferraguti F. (2016). Combined Optogenetic and Freeze-fracture Replica Immunolabeling to Examine Input-specific Arrangement of Glutamate Receptors in the Mouse Amygdala. *J. Vis. Exp*. 2016:53853. 10.3791/53853 27167567 PMC4941933

[B66] Segal M. (2017). Dendritic spines: morphological building blocks of memory. *Neurobiol. Learn. Mem.* 138 3–9. 10.1016/j.nlm.2016.06.007 27311757

[B67] Smith P. H. Ulrich D. J. Manning K. A. (2019). Evaluation of medial division of the medial geniculate (MGM) and posterior intralaminar nucleus (PIN) inputs to the rat auditory cortex, amygdala, and striatum. *J. Comp. Neurol.* 527 1478–1494. 10.1002/cne.24644 30689207 PMC6690052

[B68] Strobel C. Marek R. Gooch H. M. Sullivan R. K. Sah P. (2015). Prefrontal and auditory input to intercalated neurons of the amygdala. *Cell. Rep.* 10 1435–1442. 10.1016/j.celrep.2015.02.008 25753409

[B69] Szoboszlay M. Kirizs T. Nusser Z. (2017). Objective quantification of nanoscale protein distributions. *Sci Rep.* 7:15240. 10.1038/s41598-017-15695-w 29127366 PMC5681686

[B70] Tanaka J. Matsuzaki M. Tarusawa E. Momiyama A. Molnar E. Kasai H. (2005). Number and density of AMPA receptors in single synapses in immature cerebellum. *J. Neurosci.* 25 799–807. 10.1523/JNEUROSCI.4256-04.2005 15673659 PMC6725634

[B71] Tarusawa E. Matsui K. Budisantoso T. Molnár E. Watanabe M. Matsui M. (2009). Input-specific intrasynaptic arrangements of ionotropic glutamate receptors and their impact on postsynaptic responses. *J. Neurosci.* 29 12896–12908. 10.1523/JNEUROSCI.6160-08.2009 19828804 PMC6665298

[B72] Turner B. H. Herkenham M. (1991). Thalamoamygdaloid projections in the rat: a test of the amygdala’s role in sensory processing. *J. Comp. Neurol.* 313 295–325. 10.1002/cne.903130208 1765584

[B73] Wu D. Bacaj T. Morishita W. Goswami D. Arendt K. L. Xu W. (2017). Postsynaptic synaptotagmins mediate AMPA receptor exocytosis during LTP. *Nature* 544 316–321. 10.1038/nature21720 28355182 PMC5734942

[B74] Xie M. J. Ishikawa Y. Yagi H. Iguchi T. Oka Y. Kuroda K. (2019). PIP 3-Phldb2 is crucial for LTP regulating synaptic NMDA and AMPA receptor density and PSD95 turnover. *Sci. Rep.* 9:4305. 10.1038/s41598-019-40838-6 30867511 PMC6416313

[B75] Yeh S. Mao S. Lin H. Gean P. (2006). Synaptic expression of glutamate receptor after encoding of fear memory in the rat amygdala. *Mol. Pharmacol.* 69 299–308. 10.1124/mol.105.017194 16219906

